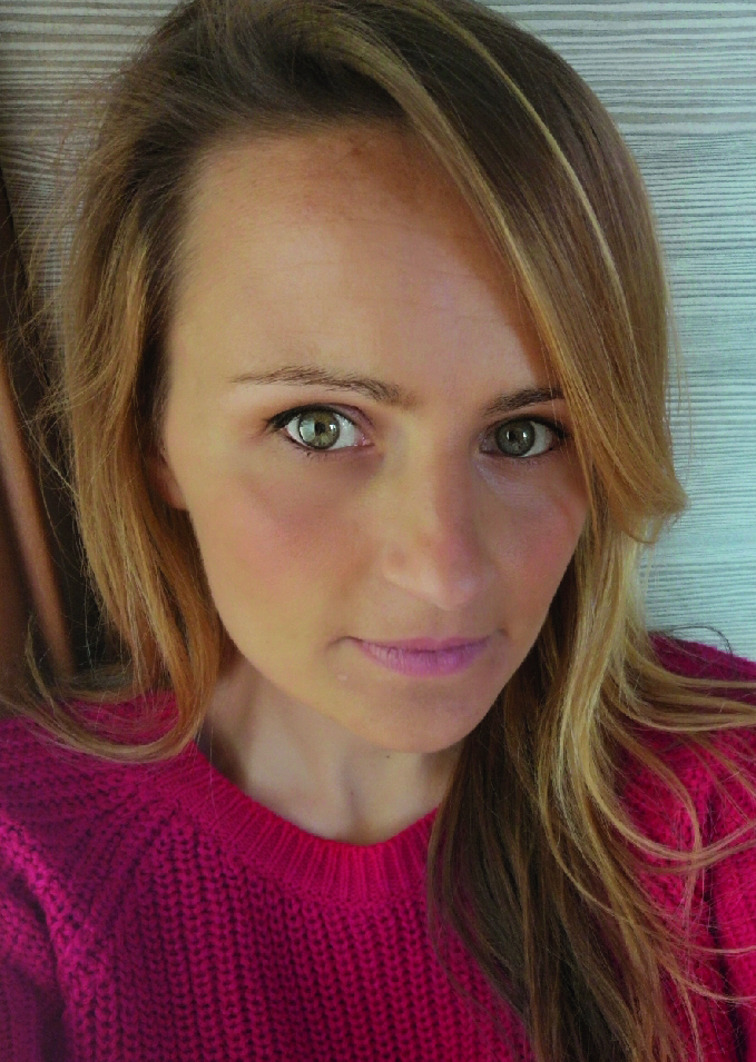# Beyond the Toll‐Like Receptor 4. Structure‐Dependent Lipopolysaccharide Recognition Systems: How far are we?

**DOI:** 10.1002/cmdc.202400780

**Published:** 2025-01-15

**Authors:** Stefania De Chiara, Luca De Simone Carone, Roberta Cirella, Emanuela Andretta, Alba Silipo, Antonio Molinaro, Marcello Mercogliano, Flaviana Di Lorenzo

**Affiliations:** ^1^ Department of chemical sciences University of Naples Federico II via Cinthia 4 80126 Naples Italy; ^2^ CEINGE, Istituto di Biotecnologie avanzate Via Gaetano Salvatore, 486 80131 Naples Italy; ^3^ Department of Chemistry School of Science Osaka University 1-1 Osaka University Machikaneyama Toyonaka Osaka 560-0043 Japan

**Keywords:** Lipopolysaccharide, Innate Immunity, Structure-activity relationships, Medicinal chemistry, Immunology

## Abstract

With an enormous potential in immunology and vaccinology, lipopolysaccharides (LPSs) are among the most extensively studied bacteria‐derived molecules. LPS centered studies are countless, and their results reverberate in all areas of the life sciences, including chemistry, biology, genetics, biophysics, and medicine. Most of these research activities are focused on the LPS‐induced immune response activation by means of Myeloid Differentiation protein‐2/Toll Like Receptor 4 (MD‐2/TLR4) complex, which currently is the most largely explored LPS sensing pathway. However, the enormous structural variability of LPS allows interactions with numerous other receptors involved in a wide range of equally important immunological scenarios. In this review, we explore these additional LPS recognition systems, which operate within interconnected signaling cascades, highlighting their role in maintaining physiological homeostasis and their involvement in the development of severe human diseases. Understanding these pathways, their interconnections, and the crosstalk between them and TLR4/MD‐2 is essential for guiding the development of pharmacologically active molecules that could specifically modulate the inflammatory response, paving the way to new strategies for combating immune‐mediated diseases and resistant infections.

## Graphical abstract

This review goes beyond the famous MD‐2/TLR4‐mediated recognition of LPS, pointing the attention on other immune detection systems and their impact on human physiology and pathology. Understanding these additional pathways is key for the development of pharmacologically active molecules to modulate the immune response and to conceive new strategies to combat immune‐mediated diseases and resistant infections.

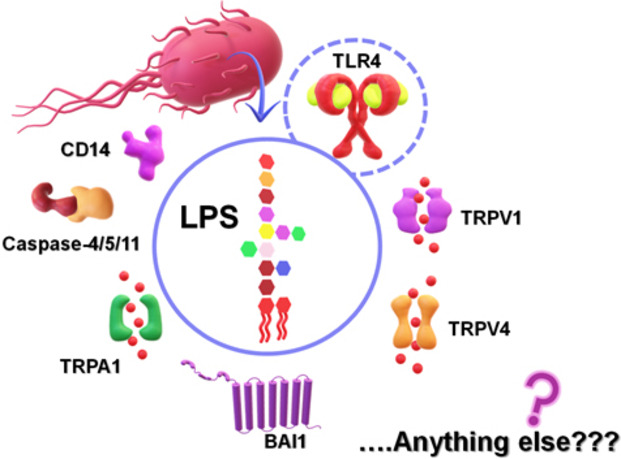

## 1 Introduction

Lipopolysaccharide (LPS) is the main constituent of the outer membrane of Gram‐negative bacteria. It structurally comprises a saccharide portion and a lipid portion (lipid A), where the former can be further distinguished in two moieties, i. e. the core oligosaccharide (core OS) and the O‐polysaccharide chain (or O‐Antigen).^[1]^ An LPS built up of these three domains is termed smooth‐type LPS (S‐LPS), whereas when the polysaccharide region is absent or truncated, it is called rough‐type LPS (R‐LPS) or lipooligosaccharide (LOS). Being exposed on the cell surface, LPS molecules are involved in a plethora of crucial host–bacterium interaction events such as adhesion, colonization, symbiosis, tolerance, and virulence.[^2^, ^3^] However, the LPS is certainly widely acknowledged as a potent stimulator of the immune response in a broad range of eukaryotic hosts, ranging from insects to humans.[^1^, ^2^, ^3^] Acting as a microbe associated molecular pattern (MAMP), it is detected in a structure‐dependent manner by specific receptors of the host innate immune system, known as Pattern Recognition Receptors (PRRs). Among these PRRs, the receptorial complex MD‐2/TLR4, which is expressed on several immune and non‐immune cells, perceives the LPS and it has been considered the only sensor of this glycoconjugate for a long time.[^4^, ^5^] Nevertheless, it is now well established that LPS is a versatile molecule able to behave as an actor, if not as the protagonist, on many (immunological) stages.[^1^, ^6^] A series of host molecules have shown the ability to detect LPS, thus disclosing a complex and assorted repertoire of receptors that points out the relevance of host–microbe interactions mediated by LPS across all the kingdoms of life.^[7]^ Therefore, the story of LPS recognition by the immune system still has a long story to be developed ahead.

A detailed description of the TLR4‐dependent pathway(s) is beyond the scope of this review. Nevertheless, given its capacity to potently stimulate an immune inflammatory response following its recognition by this receptorial complex, it is not surprising that most of the LPS centered studies have focused their attention and efforts on deciphering the molecular details of this interaction. As a matter of fact, acquiring insights into how this binding occurs lays the foundation for modulating TLR4 activation and signaling, which is an urgent need from both a pharmacological and clinical point of view.^[2]^ Indeed, on one hand, finely tuning the immune response is useful for the development of vaccine adjuvants; on the other hand, inhibition of TLR4 is considered a therapeutic approach to detrimental Gram‐negative infections.[^8^, ^9^, ^10^] Consequently, a multitude of studies have been devoted to understanding how the different TLR4‐mediated immune responses are connected to the diverse LPS structures, whose activity spans from potently pro‐inflammatory (TLR4 agonists) to anergic and even inhibitory (TLR4 antagonists), and all depending on the chemical features of LPSs.^[1]^

**Figure 1:**
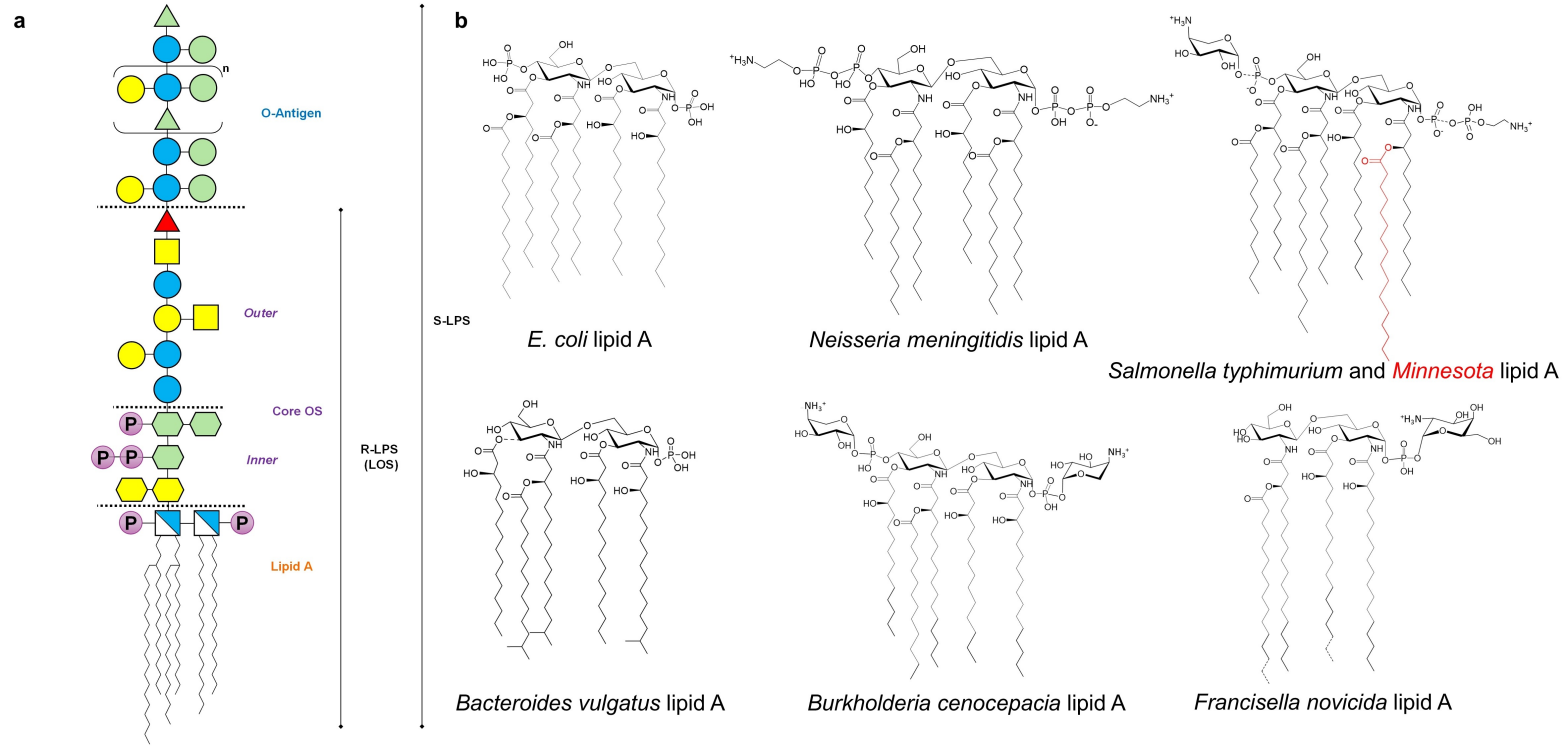
(**a**) Cartoon describing a general architecture of an LPS. The structure is hypothetical and has been created for demonstration purposes only. Zigzag lines in the lipid A represent fatty acids. (**b**) Structural heterogeneity in the lipid A. *E. coli* lipid A, which has the prototypical TLR4 agonistic structure, is made up of a *bis*‐phosphorylated glucosamine disaccharide backbone decorated by six acyl chains with a 4+2 distribution [14 : 0 (3‐OH) as primary, 14 : 0 and 12 : 0 as secondary fatty acids]. *Neisseria meningitidis* lipid A typically presents a 3+3 symmetry of the acyl moieties with respect to the lipid A sugar backbone, and both phosphate groups are substituted by 2‐aminoethylphosphate groups, thus giving rise to 2‐aminoethylpyrophosphate units. *Salmonella Minnesota* displays a lipid A with a *bis*‐phosphorylated diglucosamine backbone carrying 7 fatty acids that are distributed according to a 5+2 asymmetry, while *S. typhimurium* is typically hexa‐acylated with a 4+2 distribution of the acyl chains. However, in response to environmental conditions, also *S. typhimurium* lipid A can be hepta‐acylated. Both *Salmonella* lipid As can be not‐stoichiometrically substituted by L‐aminoarabinose and/or 2‐aminoethylphosphate on the phosphate moieties. *Bacteroides vulgatus* lipid A, which is a weak TLR4 agonist, is penta‐or tetra‐acylated and typically carries only one phosphate, and odd numbered and branched acyl chains. *Burkholderia cenocepacia* typically expresses a mixture of tetra‐ and penta‐acylated lipid A species carrying 16 : 0 (3‐OH) and 14 : 0 (3‐OH) as primary, and 14 : 0 as secondary fatty acids, plus L‐aminoarabinose units on one or both the phosphate groups. *Francisella novicida* lipid A has four acyl chains of 16–18 carbons in length and only one phosphate group on the reducing glucosamine unit. The latter can be further modified by the addition of galactosamine onto the phosphate moiety. Dotted lines indicate non‐stoichiometric substitution.

**Figure 2:**
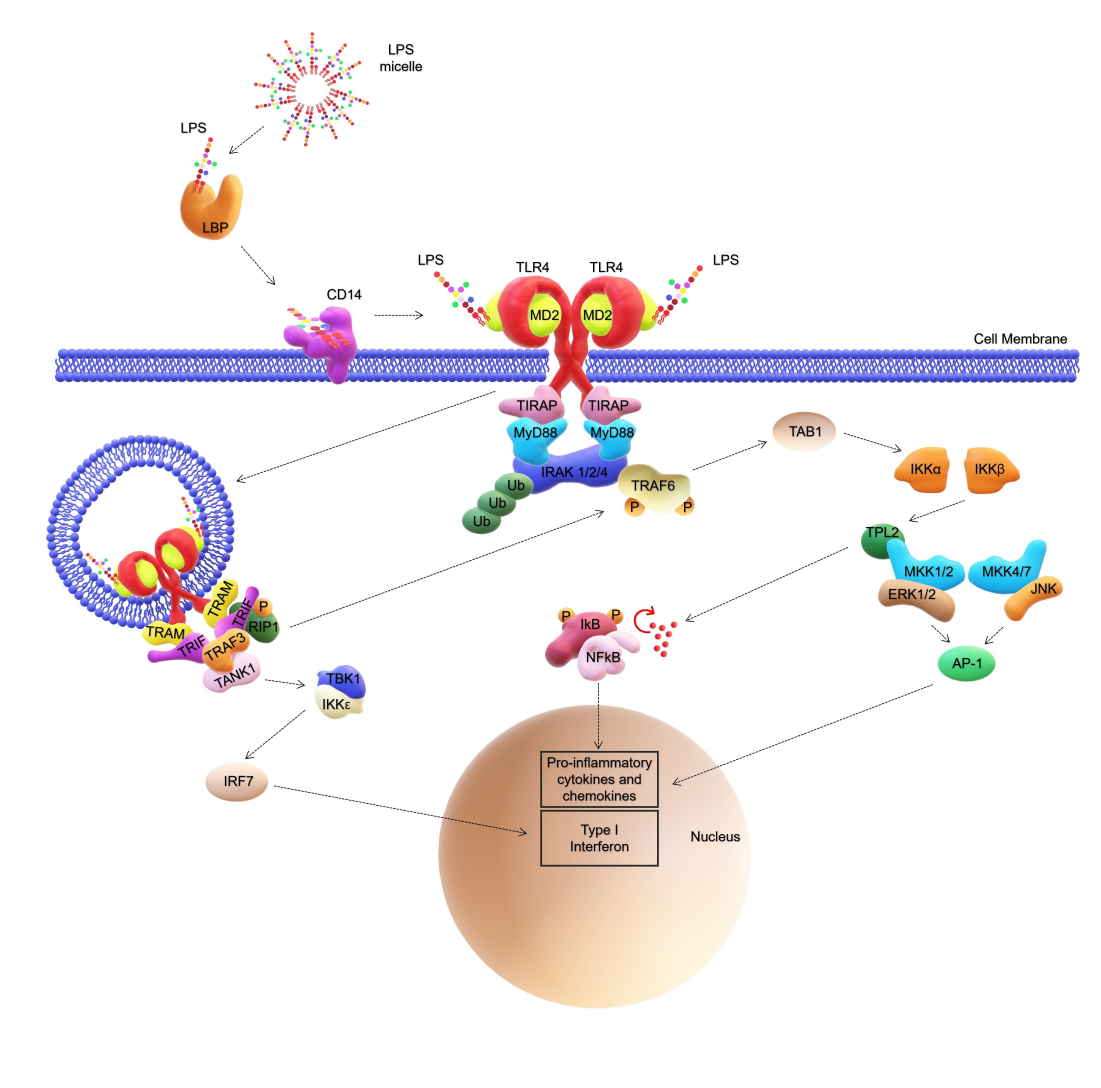
Simplified unscaled representation of MD‐2/TLR4 complex signaling induced by extracellular LPS. LBP extracts a single molecule of LPS from the micelle to deliver it to the MD2/TLR4 complex via CD14. The interaction with LPS results in the dimerization of the receptor, thereby activating two distinct signal transmission pathways. The first is the “Myddosome,” also known as the MyD88‐dependent pathway, which occurs at the cell surface. The second is the “Triffosome,” or TRIF‐dependent pathway, which originates from the endosome. Abbreviations: LPS, Lipopolysaccharide; LBP, Lipid binding protein; CD14, Cluster of differentiation 14; TLR4, Toll‐like receptor 4; MD‐2, Myeloid differentiation factor‐2; MyD88, Myeloid differentiation primary response 88; TRIF, Toll/IL‐1R domain‐containing adaptor‐inducing IFN‐β; TIRAP, TIR‐domain‐containing adapter protein; IRAK, IL‐1 receptor‐associated kinase; TRAF, TNF receptor‐associated factor; TAB1, TAK1‐binding protein; TANK1, TRAF family member associated NF‐κB activator1; TBK1, TANK‐binding kinase 1; TPL, Tumor progression locus 2; AP1, Activator protein 1; MKK, Mitogen‐activated protein kinase; TRAM, TRIF‐related adaptor molecule; IκB, NF‐κB inhibitor; NF‐κB, Nuclear factor kappa‐light‐chain‐enhancer of activated B cells; JNK, C‐Jun N‐terminal kinase; RIP1, Receptor‐interacting protein; IKK, IKβ kinase; IRF7, IFN‐regulatory factor 7; ERK, Extracellular signal‐regulated kinase; Ub, Ubiquitination; P, Phosphate.

Generally speaking, the main contributor to this behavior is the lipid A moiety which is specifically recognized by the MD‐2/TLR4 complex in a structure dependent manner.^[11]^ In this frame, the preparation of synthetic derivatives greatly contributed to the unequivocal determination of the lipid A minimal structure that is essential for the TLR4‐immunostimulating action of an LPS. Briefly, full TLR4 agonistic activity is observed for a lipid A molecule made up of two (D‐*gluco*‐configured) hexosamine units, two phosphate groups and six fatty acids (i. e. *bis*‐phosphorylated hexa‐acylated lipid A) with a specific chain length, and a non‐symmetrical distribution with the respect to the disaccharide backbone.^[1]^ The *bis*‐phosphorylated hexa‐acylated lipid A from *E. coli* (Figure 1), as example, is considered the prototypical TLR4 agonistic structure,[^1^, ^12^] as it has the strongest immunostimulant capacity in humans, and therefore the highest pro‐inflammatory cytokine‐inducing activity.^[13]^ In contrast, hypo‐acylated lipid As (i. e. tetra‐ and some penta‐acylated) lack or exert a poor activity on human immune cells (Figure 1).[^14^, ^15^] Needless to say that any alteration of the lipid A architecture potentially affects the global TLR4 agonistic efficiency of an LPS, which in turn also is strongly influenced by receptor species‐specific sensitivity.[^1^, ^2^, ^3^, ^14^] Two are the intracellular pathways activated by lipid A when sensed by TLR4: the so called “Myddosome” (or MyD88‐dependent pathway)^[16]^ that occurs at the cell surface and the “Triffosome” (or TRIF‐dependent pathway), which takes place within the endosomes (Figure 2).^[17]^ Both pathways are competitive and mutually exclusive, and lead to cytokine production that when excessive can lead to sepsis and septic shock.

From a clinical and pharmacological perspective, these pathways involving the MD‐2/TLR4 complex are of undeniable importance and worthy of being researched and still studied further. However, a more comprehensive understanding of the LPS‐mediated immune framework and the possible dialogue between all its receptors is compulsory to be able in future to come out with therapeutic strategies against bacterial infections and/or immune inflammatory reactions. In the last two decades, in fact, several LPS recognition systems have been discovered thus broadening our knowledge of the evolutionary “arms race” of the human host against bacterial intruders which, in turn, have developed elaborated strategies to evade immune vigilance by altering the chemistry of their LPS. In this review we provide an overview of the current state of the art and recent developments in this still understudied research area by casting an eye on the existing gaps and the urgent need for advances in this field. Our intention is to boost the interest towards research activities devoted to expanding knowledge of these still uncovered LPS sensing pathways, their potential crosstalk and effects on human physiology/pathology. We aim to draw attention to the lack of structure to function studies on LPS recognition systems beyond TLR4, an aspect that remains largely unexplored. Addressing these gaps will pave the way for developing targeted therapies to modulate the inflammatory response, to produce next‐generation vaccines, and therefore to improve translational research for the benefit of human health. We will discuss the role of extracellular receptors of LPS, such as CD14, and intracellular ones, such as caspases and components of the non‐canonical inflammasome. Likewise, the activation of less explored but not less important pathways, such as those involving Transient Receptor Potential (TRP) channels, Brain‐specific Angiogenesis Inhibitor 1 (BAI1) or High‐Mobility Group Box 1 (HMGB1) and even carbohydrate binding proteins, known as lectins, will also be debated. For the sake of simplicity, each receptor will be discussed separately, although it is clear that they are part of interconnected signaling pathways and work together within an extremely complex immunological context. Finally, when reported in literature, the impact of the chemical structure of LPS and its single moieties in the activation of these pathways will also be discussed; likewise, when indicated in the related study, the bacterial species and strain will be reported, otherwise, only the general term LPS will be employed.

## 2 The TLR4‐Independent Role of CD14 in LPS‐Triggered Signaling Pathways

### 2.1 General Overview of CD14 as a co‐Receptor of TLR4

CD14 is a glycoprotein that can be distinguished into two forms: membrane (mCD14) or soluble (sCD14). In particular, mCD14 is anchored to the plasma membrane via glycosylphosphatidylinositol (GPI) moiety.^[18]^ In contrast, sCD14 can be found in both extracellular fluids and blood.[^18^, ^19^, ^20^] CD14 expression has been detected both in immune cells, such as macrophages, monocytes, dendritic cells (DCs) and granulocytes, and in non‐immune cells including hepatocytes, spermatozoa, smooth muscle cells, pancreatic islet cells and adipocytes.^[18]^ Both CD14 forms have been extensively studied as key molecules involved in the TLR4‐mediated response to LPS as they are able to bind single monomers of this glycoconjugate. In fact, LPS is released in serum by bacteria as micelles which are first bound by Lipid Binding Protein (LBP), then, CD14 physically interacts with LBP‐LPS aggregates and extracts the LPS monomers.[^1^, ^18^, ^21^, ^22^, ^23^] LPS in complex with CD14 is then transferred to the ectodomain of the MD‐2/TLR4 which dimerizes with another MD‐2*/TLR4* thus forming the M‐shaped LPS/MD‐2/TLR4 dimer that in turn leads to the initiation of the Myddosome signaling cascade (Figure 2).^[1]^ However, CD14 was also shown to promote TLR4 endocytosis, playing an important role in activation of the “Triffosome” pathway, eventually inducing interferon (IFN) expression (Figure 2).[^1^, ^24^, ^25^, ^26^] In fact, studies performed on both dendritic cells (DC) and bone marrow derived macrophages (BMDM) have reported alterations in both TLR4 endocytosis and IFN production in CD14‐deficient cells.[^27^, ^28^] Likewise, mutations occurring in CD14 LPS‐binding pocket^[29]^ were shown to reduce TLR4 internalization even in presence of other crucial molecules like LBP.^[28]^

**Figure 3:**
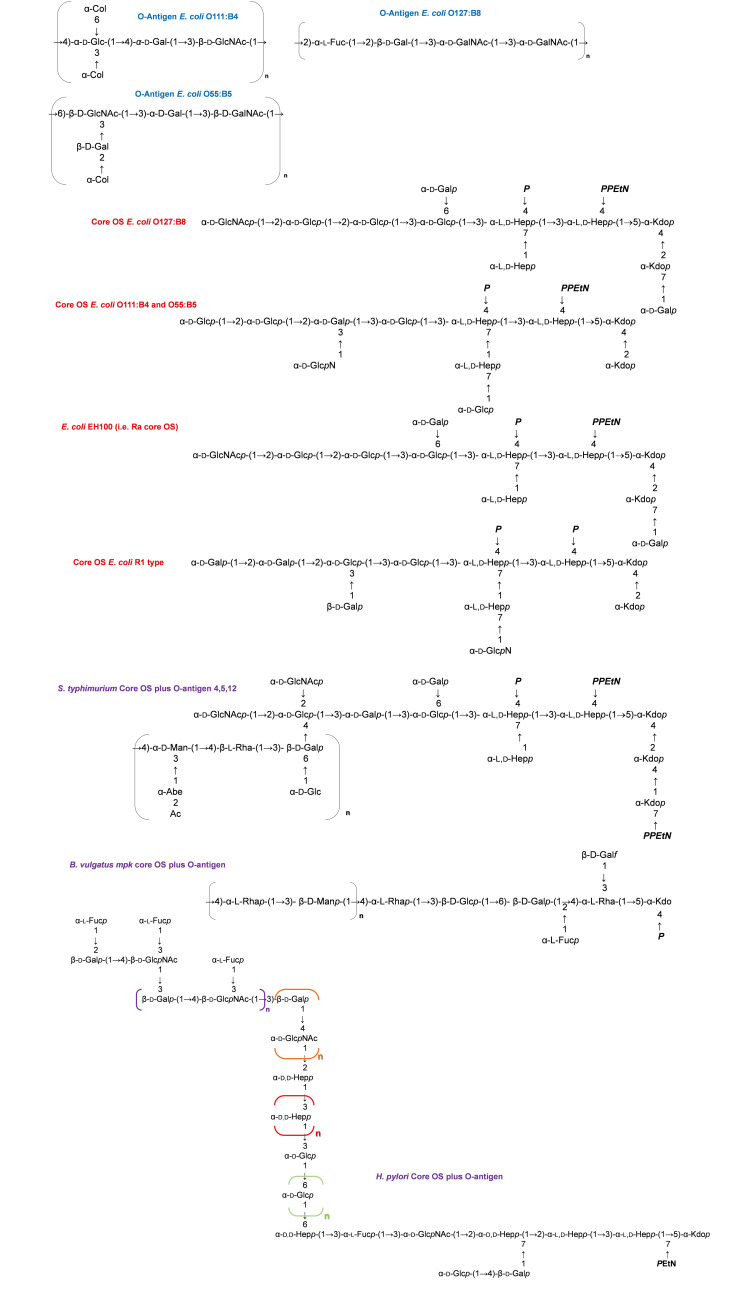
The diversity of LPS chemistry. The structures of the O‐antigen and the core OS of the main *E. coli* and *Salmonella* strains whose LPS has been used in the studies mentioned in this review have been reported in the figure. The structure of other chemically different LPSs, such as from *Bacteroides vulgatus* mpk and *Helicobacter pylori*, have been also reported and mentioned in the text. Abe: abequose; Col: colitose; Kdo*p*, 3‐deoxy‐α‐D‐*manno*‐oct‐2‐ulopyranosonic acid; L,D‐Hep*p*: L‐*glycero*‐D‐*manno*‐heptopyranose; D,D‐Hep*p*: D‐*glycero*‐D‐*manno*‐heptopyranose; D‐Glc*p*: D‐glucopyranose; D‐Gal*p*: D‐galactopyranose; D‐Gal*f*: D‐galactofuranose; D‐Glc*p*NAc: *N*‐acetyl‐D‐glucosamine (2‐acetamido‐2‐deoxy‐D‐glucopyranose); D‐Gal*p*NAc: *N*‐acetyl‐D‐galactosamine (2‐acetamido‐2‐deoxy‐D‐galactopyranose); D‐Man*p*: D‐mannopyranose; L‐Fuc*p*: L‐fucopyranose; *P*EtN: 2‐aminoethylphosphate; Ac: acetyl group; *P*: phosphate. *p* and *f* indicate the pyranose and furanose form, respectively.

Still limited data has been reported to date about the structure‐dependent capability of CD14 to detect and bind LPS. In this frame, it is worth mentioning an interesting study by Albright *et al*.^[30]^ on the R‐LPS of *E. coli* WBB06, which is made up of the sole lipid A and two Kdo units, i. e. the so‐called Re form (Figure 3). The authors demonstrated that the regions involved in the interaction between sCD14 and *E. coli* WBB06 R‐LPS are located in the lower portions of the sugar headgroup and in the upper half of the acyl chains at the 2‐, 2′‐, and 3′‐positions that are proximal to 1‐ and 4′‐phosphate ends.^[30]^ Furthermore, phosphate groups were found to play a significant role since their absence determined the lack of interaction between inner core Kdo2‐Lipid A and CD14. Based on these observations, the authors suggested that the flexibility of the hydrophilic rim of CD14 may allow it to interact with structurally diverse sugar moieties, and concurrently, the rigid but large nature of the hydrophobic pocket would enable the accommodation of ligands, such as lipid A, with structural variations in their hydrophobic portion. Nevertheless, additional structure‐based studies are required to fully appreciate the mode of LPS recognition by CD14 and how this changes on the basis of LPS chemical variations. Overall, it is known that CD14 is able to recognize LPS molecules according to their “rough” (R‐LPS) or “smooth” (S‐LPS) nature. In this regard, Gangloff *et al*.^[31]^ evaluated the role of CD14 in the specific recognition of LPS, by testing S‐LPS, R‐LPS, and various partial forms of LPS, such as lipid A or phosphate‐lacking LPS, on murine CD14(+/+) and CD14(−/−) macrophages.^[31]^ Of note, CD14‐expressing macrophages were more reactive to S‐LPS molecules than CD14‐deficient macrophages. For example, S‐LPS from *Salmonella* (Figure 3) induced a more potent stimulation of CD14(+/+) macrophages than the R‐LPS from *Neisseria meningitidis*.^[31]^ Moreover, the stimulation with S‐LPS from *E. coli* 015 or with the R‐LPS from *E. coli* EH100 and *E. coli* F515 (Re LPS form) (Figure 3) proved that the absence or truncation of the O‐Antigen moiety results in a decreased response to LPS in CD14(+/+) macrophages while it increases in CD14(−/−) ones.^[31]^ Likewise, the presence of phosphate groups was also shown to be crucial as their removal resulted in a reduced response in CD14(+/+) macrophages while increased in CD14(−/−) macrophages. Therefore, and intriguingly, these studies emphasize the capability of CD14 to distinguish between LPS displaying different chemical structures.^[31]^ However, the fact that they only examine murine CD14 behavior, which may differ from its human counterpart, remains a limitation in this research field. As a matter of fact, compared with mouse CD14, human CD14 displays an expanded pocket and alternate rim residues, which likely play key roles in LPS binding and immune response activation.^[32]^ Furthermore, although chemically distinct LPSs have been analyzed, the significant diversity in both the sugar and lipid components of *Salmonella*, *Neisseria*, and *Escherichia* LPS (Figure 3) makes it challenging to gain meaningful insights into the molecular mechanisms by which murine CD14 recognizes these different LPS structures.

### 2.2 TLR4‐Independent Mechanisms Played by CD14

**Figure 4:**
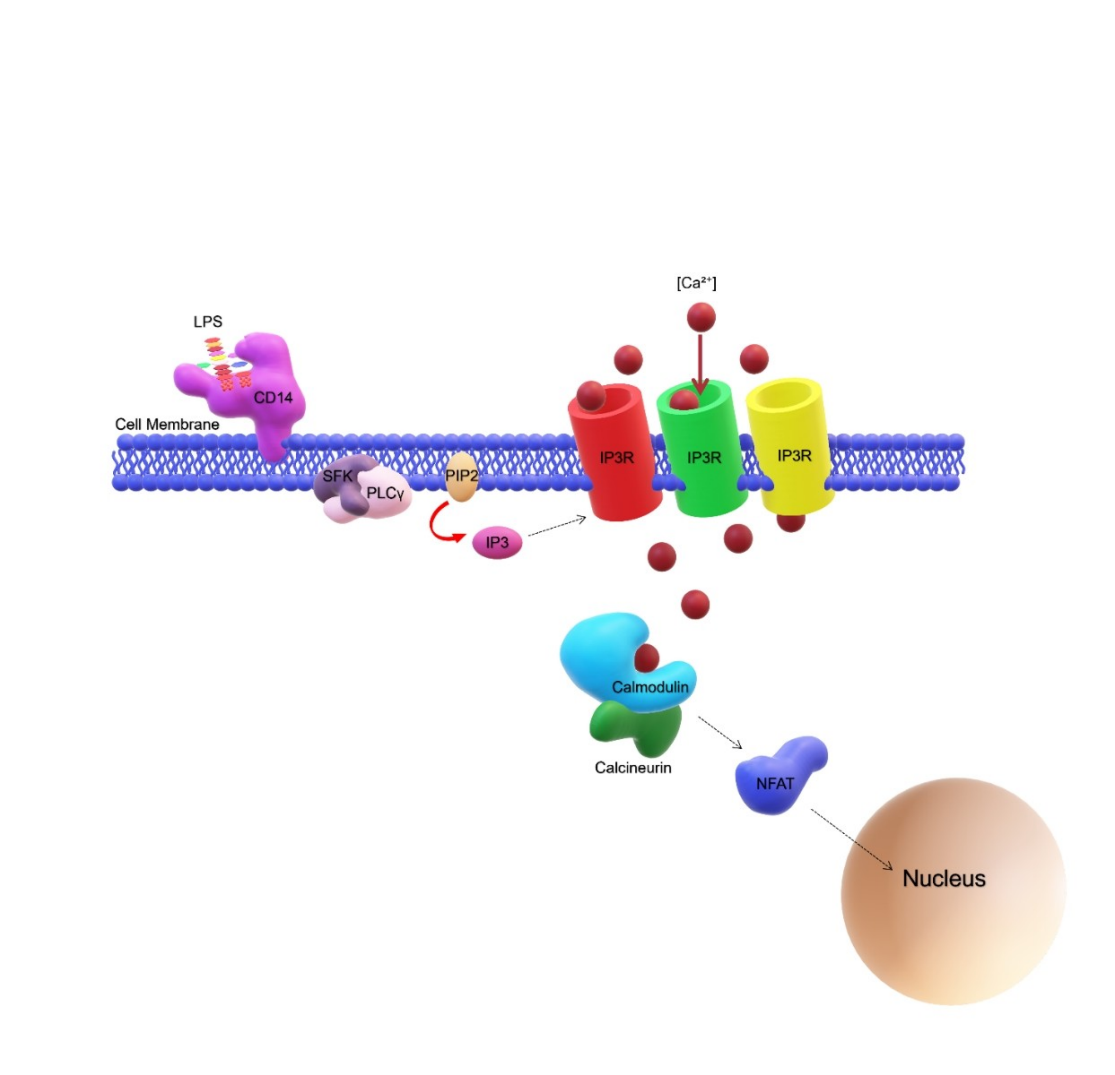
Unscaled representation of CD14 autonomous signaling pathway leading to activation of members of the NFAT family of transcription factors. CD14 promotes the activation of SFK and PLCγ, that are responsible for the conversion of PIP2 into IP3. IP3 binds to IP3 receptors, causing extracellular calcium entry and activating calcineurin‐regulated translocation of NFAT to the nucleus. Abbreviations: LPS, Lipopolysaccharide; CD14, Cluster of differentiation 14; PLCγ, Phospholipase Cγ; SFK, Src family kinase; NFAT, Nuclear factor of activated T cells; PIP2, Phosphatidylinositol 4,5‐bisphosphate; IP3, Inositol triphosphate; IP3R, IP3 receptors.

Recent studies in murine have demonstrated the existence of a CD14‐dependent LPS recognition pathway that is completely independent of TLR4 activation and that selectively affects the production of inflammatory mediators and the cell survival. Following LPS binding, in fact, mCD14 on plasma membrane of DCs activates Src family kinase (SFK) and phospholipase Cγ2 (PLCγ2).^[33]^ The latter then converts phosphatidylinositol 4,5‐bisphosphate (PIP2) to inositol triphosphate (IP3), which then binds IP3Rs, resulting in an extracellular calcium influx and subsequent calcineurin‐dependent translocation of nuclear factor of activated T cells (NFAT) into the nucleus (Figure 4).^[1]^ CD14‐dependent NFAT activation in DCs results in apoptosis of terminally differentiated DCs. Therefore, blocking this pathway entails prolongation of DCs survival and enhancement of their T‐cell priming capability.^[33]^ This implies that the termination of an activated immune response requires the CD14‐dependent NFAT pathway in DCs. As such, modulation of the CD14 is vital for preventing autoimmunity.[^33^, ^34^, ^35^] By contrast, macrophages when stimulated with S‐LPS from *E. coli* do not display calcium mobilization and NFAT activation,^[21]^ consequently, they do not experience decreased survival rates upon LPS stimulation,^[36]^ likely due to differences in the distribution and/or expression of receptors involved in calcium mobilization. As demonstrated by Zanoni *et al*.^[35]^ CD14‐dependent NFAT pathway is also involved in the regulation of immune and inflammatory response.^[35]^ In an inflammatory context, *in vivo* LPS administration induces formation of skin edema, which is one of the first steps in the initiation of an inflammatory process and consists in the local accumulation of inflammatory mediators. This phenomenon occurs through a mechanism regulated by DCs and strongly dependent on CD14‐NFAT activation.^[37]^ Briefly, after stimulation of DCs with LPS, the consequent CD14‐dependent NFAT activation promotes transcription of *Ptges‐1* that codes a protein called microsomal PGE synthase‐1 (mPGES‐1).[^35^, ^37^] mPGES‐1 in turn leads to the release of prostaglandin E_2_ (PGE_2_) that sustains the formation of edema at the inflammatory site.[^35^, ^38^, ^39^] However, it is worth noting that, to the best of our knowledge, these studies were performed by using several LPSs, i. e. S‐LPS from *E. coli* O55 : B5, O111 : B4, *S. typhimurium*, and R‐LPS from *E. coli* R515 (Re form) and its lipid A portion (Figures 1 and 3), and therefore only a strong pro‐inflammatory LPS chemistry was analyzed.^[37]^ As stated above, activation of the NFAT pathway for edema formation has been observed predominately/exclusively in DCs, in accordance to previous data pointing that the LPS/CD14/NFAT pathway is not active in macrophages.[^1^, ^37^] By contrast, it has been shown that in alveolar macrophages, LPS recognition via CD14 results in activation of the calcium channel P2X7R with the consequent induction of calcium influx and ATP depletion.^[40]^ This leads to necrosis and IL‐1α release which stimulates endothelial cell activation consenting neutrophil recruitment and supports LPS‐induced acute lung injury.^[40]^

Given the peculiar capability of DCs to involve LPS/CD14/NFAT pathway, which is not observed in macrophages, it would be extremely interesting to evaluate whether different LPS structures distinctly affect the activation of the NFAT pathway and how this process varies across different immune cell types and species. In this context, a notable study by Marongiu *et al*. (2021)^[41]^ examined whether the signaling pathway responsible for LPS‐induced NFAT activation in murine DCs is conserved in humans. Their findings revealed that in human CD1c+CD14+DCs, exposure to *E. coli* S‐LPS triggers NFAT activation, a process regulated by inositol 1,3,4,5 tetrakisphosphate (IP4), which controls calcium mobilization. By contrast, as for the LPS structure‐dependency of this activation, literature is still very limited both in murine and human models and mostly relies on an elegant study by Zanoni *et al*.^[42]^ that compared the effect of R‐LPS (*E. coli* R515, i. e. Re LPS) and S‐LPS (*E. coli* 055 : B5) (Figure 3) in inducing MyD88‐dependent, TRIF‐dependent and NFAT pathways in murine DCs. The two forms of LPS exhibited similar efficiency in activating MyD88‐dependent pathway, whereas TRIF‐dependent and NFAT pathways activation was slightly higher for S‐LPS compared to R‐LPS.^[42]^ Moreover, it was demonstrated that with respect to calcium mobilization, which in turn promotes NFAT activation, S‐LPS induced CD14‐dependent extracellular influxes, while R‐LPS triggered both intracellular calcium mobilization and extracellular calcium entry.[^33^, ^42^] Of note, the amplitude of the calcium current generated in response to R‐LPS was higher compared to S‐LPS, and in general the amplitude of calcium current generated in CD14‐deficient Bone Marrow‐Derived Dendritic Cells (BMDCs) was lower than wild‐type BMDCs.^[42]^ However, since CD14 is only able to induce an extracellular calcium entry, the authors hypothesized the existence of a still unidentified receptor required to enable the intracellular mobilization of calcium observed upon stimulation with R‐LPS.^[42]^ Investigating further these critical events will require not only considering the macromolecular nature of LPS (i. e., S‐LPS vs. R‐LPS), but also carefully examining each sugar and lipid component as potential epitopes that could influence CD14 binding and the consequent NFAT activation.

Due to the intriguing functions played by both NFAT and CD14, the development of selective inhibitors targeting the calcineurin‐NFAT pathway or CD14 and their impact on LPS‐induced inflammation or bacterial sepsis has sparked great scientific interest over the years.[^43^, ^44^, ^45^, ^46^, ^47^, ^48^] As example, *in vivo* studies demonstrated the protective role of the calcineurin‐NFAT inhibitor CP9‐ZIZIT in reducing pulmonary edema in mice affected by acute lung injury following intranasal administration of LPS.^[49]^ In contrast, Poli *et al*. observed worsening of bacterial septicemia and increased human and murine platelets aggregation upon treatment with NFAT inhibitors, thus supporting the pharmacological inhibition of the NFAT pathway in human platelets as a potential therapeutic strategy for patients with hyporesponsive platelet diseases with no currently available treatment.^[46]^ Despite these controversial results, the development or the search for CD14‐NFAT pathway inhibitors is definitely crucial to further investigate the functions of CD14 and NFAT in the inflammatory process. In this frame, recent studies have disclosed the existence of LPSs capable of only weakly or not activating TLR4, thus further supporting the hypothesis of LPS able to signal in a TLR4‐indipendent manner.[^50^, ^51^, ^52^, ^53^, ^54^, ^55^, ^56^] Among them, it is worth mentioning the LPS from the gut mutualistic bacterium *Bacteroides vulgatus* mpk which possesses an unprecedented chemical structure (Figure 3) that is able to selectively induce the production of anti‐inflammatory cytokines in human DCs and to alleviate intestinal inflammation in mice.[^51^, ^52^] Given its almost absent ability to activate TLR4, future studies should aim at dissecting the signaling pathways triggered by this and other “not inflammatory“ LPSs to evaluate any possible involvement of CD14 in this “uncommon” immunological behavior. Overall, the lack of structure‐function studies on LPS molecules that are either capable or incapable of activating the NFAT pathway via CD14 presents a significant barrier to the development of targeted inhibitors or modulators of this pathway. At the same time, it is equally important to investigate the chemistry of LPS variants that, despite not activating TLR4, still have an impact on human physiology. This dual approach is crucial for fully understanding the complex role of LPS in immune regulation.

## 3 Intracellular Sensing of LPS and The Non‐Canonical Inflammasome

In addition to binding well‐established extracellular receptors or membrane‐anchored receptors, LPS can make complexes with cytosolic structures to enhance the cellular response, as evidenced by its interaction with caspases.^[57]^ Caspases are a family of endoproteases normally associated with cell death thanks to their ability to activate the process of apoptosis, with an essential role in maintaining cell homeostasis.^[58]^ The exact process by which LPS enters the cytosol remains unclear. Actually, only a few Gram‐negative bacteria have a cytosolic phase in their life cycle, therefore intracellular release of LPS must rely on other mechanisms. One of the potential mechanisms that may facilitate this phenomenon is the formation of outer membrane vesicles (OMVs), which are membrane‐enclosed capsules containing periplasmic content, produced by both pathogenic and non‐pathogenic Gram‐negative bacteria. These can be internalized through endocytosis, delivering LPS from early endosomes to the cytosol, and activating caspases.^[59]^ Interestingly, the vesicular uptake itself is dependent on the macromolecular nature of LPS as it has been demonstrated that OMVs containing S‐LPS are more adept than those made up of R‐LPS at wrapping host membranes for endocytosis.^[59]^ Generally speaking, once accessed the cytosol, LPS is perceived by caspase‐11 (in mice) and caspase‐4 and −5 (in humans). These intracellular receptors bind LPS and lipid A with high affinity and specificity, mediating the activation of the so‐called non‐canonical inflammasome.[^60^, ^61^] The interaction between LPS and caspase‐4/5 occurs owing to a caspase recruitment domain (CARD), where residues such as leucine, isoleucine, and valine create a hydrophobic pocket ideal for interaction with the fatty acids of the lipid A portion.^[60]^ Of note, it was shown that caspase‐4 and CARD domain are able to bind large LPS micelles and to disaggregate them to small LPS/caspase‐4 complexes, which may provide the proper proximity and orientation to active caspase‐4. In contrast, charged residues, such as lysine and arginine, make ionic or hydrogen bonds with phosphate groups.^[60]^

**Figure 5:**
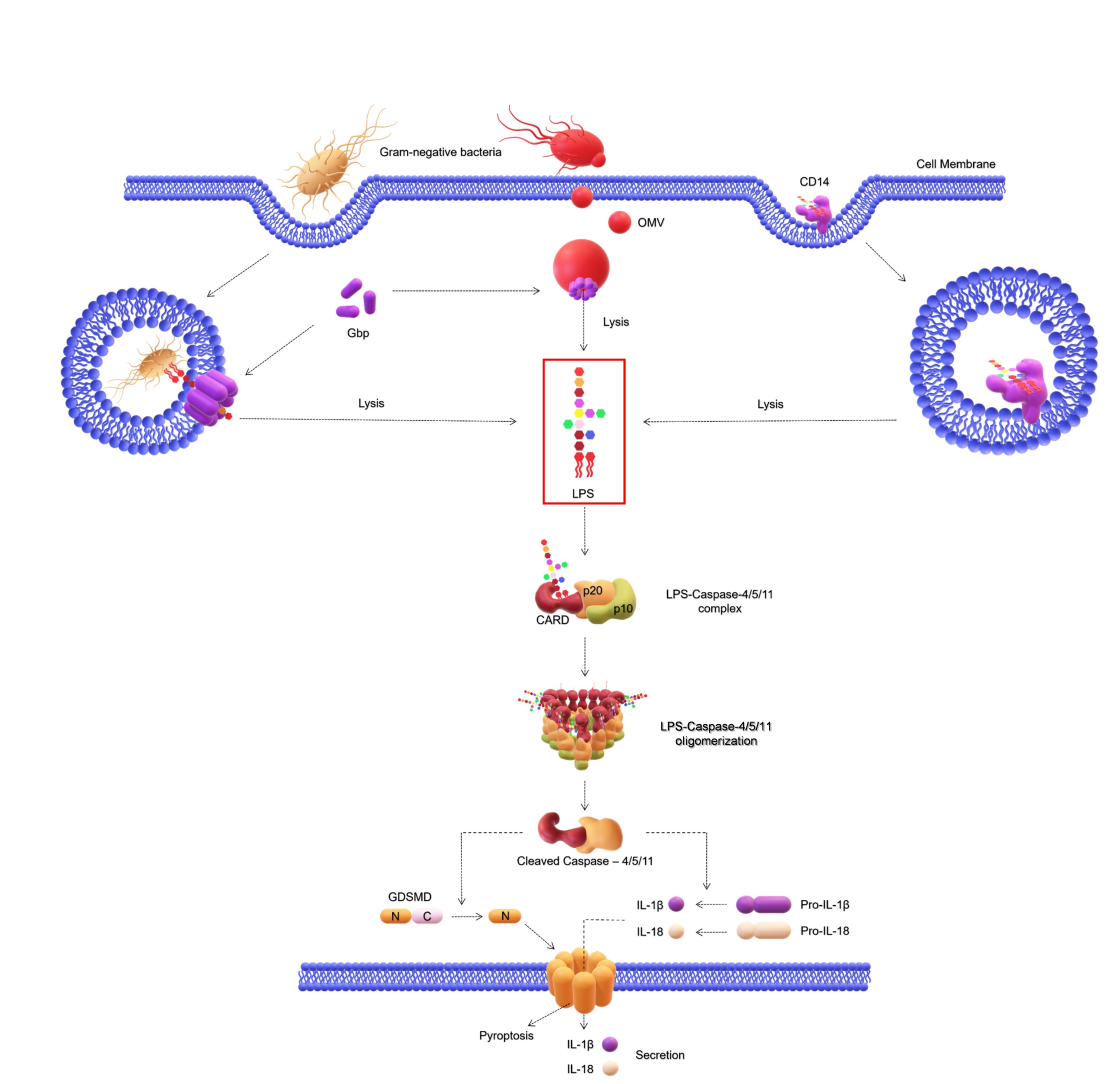
LPS can gain access to the cell in several ways. It can enter the cytosol through OMVs or via the endosome‐lysosome mechanism. The entire bacterium can be internalized and the LPS, after being extracted from the GBPs, is released in the cytosol where it is sensed by caspase‐4/5/11 via CARDs, leading to the formation of LPS‐caspase complexes. LPS can also interact with CD14, be internalized into an endosome, and then be released by lysis to reach the CARD complex. Following, GSDMD proteolysis is induced to produce N‐terminal and C‐terminal GSDMD fragments. N‐terminal GSDMD fragments generate pores in cell membranes as a result of their oligomerization which in turn induces pyroptosis. Additionally, activated non‐canonical caspase‐4 inflammasome also induces proteolytic maturation of pro‐IL‐1β and pro‐IL‐18, which are then secreted by cells through GSDMD pores. Not drawn to scale. Abbreviations: LPS, Lipopolysaccharide; OMV, Outer membrane vesicle; Gbp, Guanylate‐binding protein; CD14, Cluster of differentiation 14; CARD, Caspase recruitment domain; GSDMD, Gasdermin D; N, GSDMD N‐terminal fragment; C, GSDMD C‐terminal fragment; IL‐1β, Interleukin‐1β; IL‐18, Interleukin‐18.

Upon binding to LPS, the oligomerization of caspase‐11 and −4 monomers occurs, which in turn triggers their catalytic activity (Figure 5). The complex binds the N‐terminal portion of gasdermin D (GSDMD), which in turn detaches from the C‐terminal, binds the membrane, oligomerizes and induces the formation of pores. The presence of these pores consents the influx of calcium that leads to an increase of the internal water volume of the cell until membrane rupture with the consequent release of cytosolic content.^[62]^ Strikingly, it was also shown that the concomitant efflux of potassium through GSDMD pores provokes activation of the NLRP3/ASC/caspase‐1 inflammasome, which in turn prompts the secretion of the pro‐inflammatory cytokines IL‐1β and IL‐18.^[63]^ Following this event, cells belonging to the immune system are recalled to the site and release additional cytokines, such as IFN‐γ, which in turn regulate the production of several proteins like guanylate‐binding proteins (GBPs).^[64]^ Of the seven different types of GBPs in humans, only GBP1 was shown to bind LPS and to influence LPS‐induced signaling by modulating the induction of cytokines, chemokines, and IFN‐inducible effector molecules.[^65^, ^66^] Hence, GBP1 should be officially included in the list of molecules requiring further investigation to assess its role in regulating the LPS‐induced immune response in both health and disease.

Under the structural perspective, several studies showed that tetra‐acylated lipid A acts as a weak activator not only of MD‐2/TLR4 but also of caspase‐11.[^60^, ^67^, ^68^] In particular, Hagar *et al*. showed that caspase‐11 responds to penta‐ and hexa‐acylated lipid A from *E. coli*, whereas tetra‐acylated lipid A is not sensed, thus providing a mechanism of evasion for intracellular pathogenic bacteria, such as cytosol‐invasive *Francisella* that expresses a peculiar tetra‐acylated lipid A (Figure 3).^[67]^ By contrast, it was shown that caspase‐4 is able to perceive and actively respond to the tetra‐acylated lipid A from *Francisella novicida* (Figure 1) while escaping caspase‐11 detection in mice,^[69]^ thus suggesting a broader repertoire of LPS structures recognized by human caspases compered to mice ones. These findings further point out that a careful use of results attained from mouse models to mimic human inflammasome‐dependent responses must be done and that they should be always accompanied by in depth studies aimed at determining the molecular aspects of the intracellular recognition of LPS ad how it diverges on the basis of LPS structure and animal species. In this context, Zamyatina *et al*. provided a helpful list of structural characteristics of lipid A in relation to bacterial species known to induce caspase‐4/11‐mediated inflammasome activation.^[70]^ Briefly, caspases‐4/11 appear to be more receptive to the number of phosphate groups decorating the glucosamine backbone of lipid A than to the acylation pattern. In addition, lipid A phosphate groups chemically conjugated with positively charged appendages may be crucial for the detection of LPS by inflammatory caspases and related proteins.^[70]^ Interestingly, experiments using metabolically radiolabeled R‐ and S‐LPS from *E. coli*, which included LPS variants with different lengths of the carbohydrate portion, as well as the LPS‐Re form (Figure 3) demonstrated high affinity and selective binding of caspase‐4 to high molecular‐weight aggregates of LPS and to LPS‐rich OMVs. These findings proved a noticeably different LPS recognition properties of caspase‐4 from that of MD‐2/TLR4 and suggest that activation of caspase‐4 (and possibly −5 and −11) are mediated by interactions with activating LPS‐rich membrane interfaces rather than by LPS monomers.^[68]^

Finally, while the role of the lipid A portion in the activation of caspases seems to be rather defined, that of the carbohydrate portion is still not clear. Though, Gunther *et al*. showed that *Shigella flexneri* S‐LPS is able to block caspase activity *in vitro* and *in vivo* through a direct bind to caspases that involves the O‐antigen moiety.^[71]^ This LPS‐mediated inhibition blocks apoptosis and ensures bacterial replication and effective colonization.^[71]^ Nevertheless, studies aimed at gaining insights into if and how chemically diverse O‐antigen moieties impact on this phenomenon are still missing.

As further evidence of the complexity of LPS sensing and the intricate crosstalk among the various receptors discussed in this review, which are capable of recognizing LPS, it is worth underlining that NFAT activation is not the only TLR4‐independent process elicited by CD14 following LPS stimulation. As stated above, it is well known that CD14 facilitates the internalization of TLR4 by endocytosis,^[27]^ but recently its involvement in promoting TLR4‐independent LPS cytoplasmic localization and activation of non‐canonical inflammasome pathway has also been verified.^[72]^ *In vivo* experimental evidence in mice has demonstrated that CD14 promotes non‐canonical inflammasome pathway by causing IL‐1β and IL‐18 cytokine production and GSDMD cleavage.^[72]^ In particular, the levels of IL‐1β and IL‐18 were shown to be significantly lower in the plasma of *TLR4*−/− *CD14*−/− mice compared to the *TLR4*−/− mice after the administration of S‐LPS from *E. coli* O111 : B4 (Figure 3)^[72]^ Thus demonstrating that CD14 is an integral component also of the non‐canonical inflammasome. However, CD14‐mediated cytosolic delivery of LPS has been demonstrated only *in vivo* and not in *in vitro*, such as using BMDMs, despite these cells do express CD14.^[72]^ This implicates the involvement of a still unidentified *in vivo‐*specific actor participating in this event. Moreover, such an intriguing LPS‐mediated crosstalk between caspases and CD14 has been only investigated in murine models, therefore the role of CD14 in promoting LPS internalization and non‐canonical inflammasome activation must still be confirmed in humans.

## 4 TRP Ion Channels and LPS Recognition

TRP (Transient Receptor Potential) channels are a family of cation channels widely distributed across all human organs, such as skin, nerves, lungs, liver, and intestine, and are involved in sensory perception and both normal and pathological cell functioning.^[73]^ According to their amino acid sequence and topological differences, mammalian TRPs can be separated in seven subfamilies: TRPA (ankyrin), TRPC (canonical), TRPM (melastatin), TRPML (mucolipin), TRPN (NO‐mechano‐potential, NOMP), TRPP (polycystin) and TRPV (vanilloid).^[74]^ Due to their ability to trigger ionic changes inside and outside cells, TRP channels are involved in multiple signaling pathways, including the mitogen‐activated protein kinase (MAPK) pathway, the transforming growth factor (TGF)‐β signaling pathway, NF‐kB pathway, and the AMP‐activated protein kinase (AMPK) pathway.^[73]^ These widespread channels can detect LPS, which implies that they may couple the stimulation of sensory afferent fibers and immune responses during sepsis. Indeed, sepsis causes somatic or visceral pain, in addition to severe inflammation, and these symptoms are also associated with TRP channels. Upon their activation, in fact, an increment of intracellular calcium concentration occurs, which in turn allows the release of mediators, eventually leading to pain. However, increase of intracellular calcium content is also known to be involved in the TLR4‐dependent immune response to LPS, although the underlying mechanisms remain unclear.[^73^, ^74^]

It has been demonstrated that TRPM7 plays a role in the LPS‐induced activation of macrophages, promoting an increase in cytosolic calcium, which is critical for TLR4 endocytosis and subsequent activation of the transcription factor IRF3.^[75]^ Alternatively, calcium influx via TRPM2 plays a pivotal role in cytokine production, with intracellular levels of TRPM2 regulated by TLR4 activation and TLR4‐mediated activation of TRPC1 thus enhancing the inflammatory response.^[76]^ Taken together, this information provides evidence of the intersection between signaling pathways mediated by TLR4 and TRP. Therefore, detailed investigation of this LPS‐mediated crosstalk, the effects of TRP activation on sepsis and the immune response definitely are an intriguing and promising area of research.

**Figure 6:**
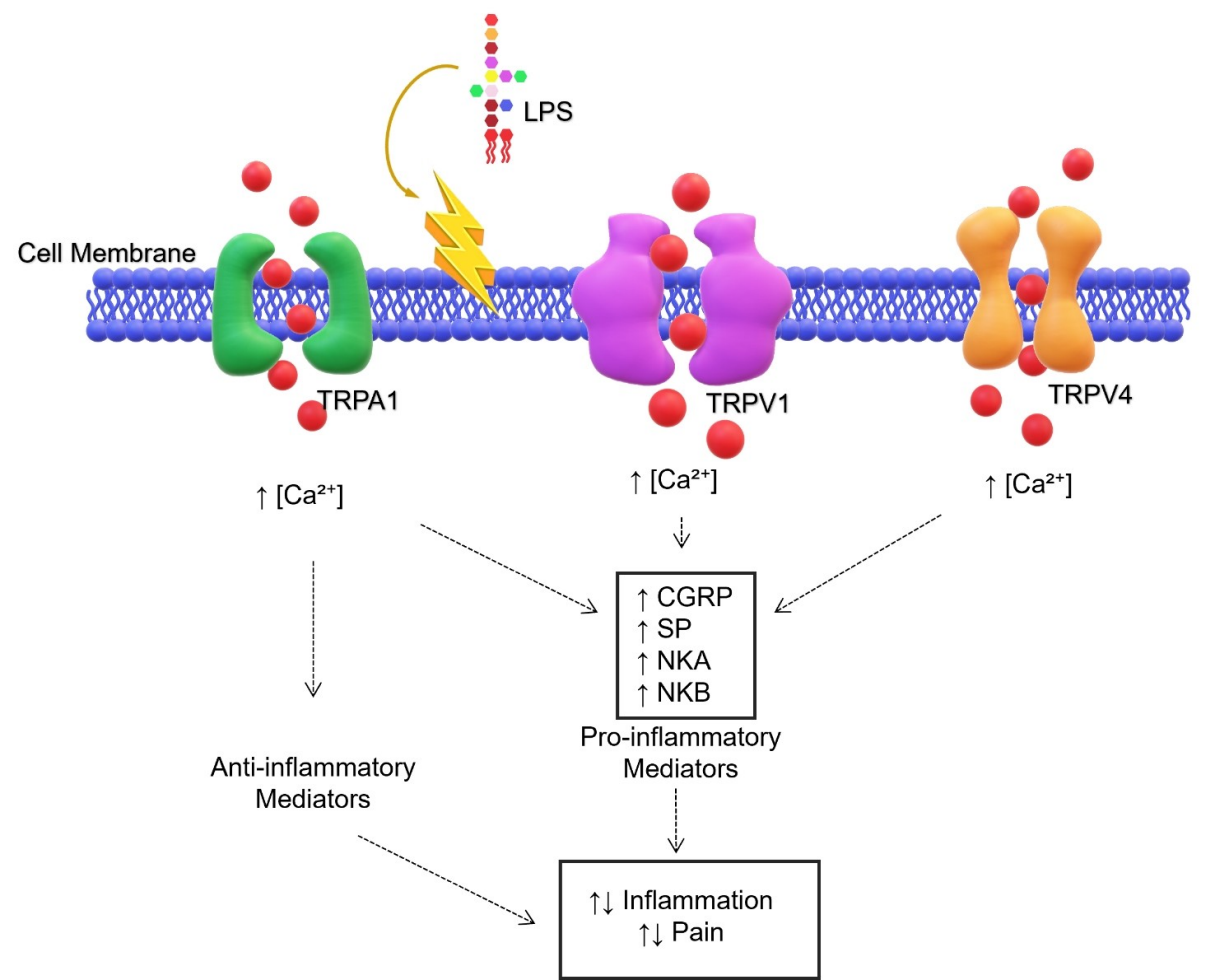
Schematic representation of three of the major transient receptor potential (TRP) channels: TRPA1 (ankyrin), TRPV1 and TRPV3 (vanilloid). TRPs, after the detection of the LPS, modulate the inflammatory response in immune cells by leading to calcium influx, which allows the production of pro‐ or anti‐inflammatory mediators. Not drawn to scale. Abbreviations: LPS, Lipopolysaccharide; CGRP, Calcitonin gene‐related peptide; SP, Substance P; NKA, Neurokinin A; NKB, Neurokinin B.

Several studies have shown that acute responses, such as hyperalgesia and tissue swelling, are mediated by TRPA1, which also works as an LPS sensor (Figure 6).[^77^, ^78^] A recent study showed that chemogenetic activation of TRPA1‐expressing vagal neurons suppresses pro‐inflammatory cytokine levels while increasing IL‐10 levels in mice treated with the S‐LPS from *E. coli* O111 : B4 (Figure 3).^[79]^ These results demonstrate the existence of information transfer from the periphery to the central nervous system via the LPS‐TRPA1 interaction. However, the exact mechanism behind LPS‐mediated activation of TRPA1 remains mostly unclear. Interestingly, Meseguer *et al*. showed that in response to LPS, and in particular to the lipid A portion, TRPA1 channels cause a TLR4‐independent increase in calcium influx in neurons that triggers neuronal excitation and sensation of pain.[^80^, ^81^] It was also demonstrated that LPS treatment, in isolated membrane patches, enhances the flow of ions across the membrane thus generating TRPA1 currents and suggesting that this channel might play a key role in sensing LPS membrane perturbations.[^7^, ^80^, ^81^, ^82^] In particular, it was shown that S‐LPS from *E. coli* exerts a stronger effect on the perturbation of plasma membrane mechanical properties than S‐LPS from *S. Minnesota*. Remarkably, this divergent behaviour has been attributed to the differences in the chemical structures of the lipid A moieties, and in particular to the different number of acyl chains decorating the lipid A of both bacteria (6 acyl chains in *E. coli* versus 7 acyl chains in *S. Minnesota*) (Figure 1).^[79]^ Actually, the hexa‐acylated lipid A from E. *coli*, which adopts a conical shape upon insertion into the membrane, might cause more alterations in the membrane than lipid A adopting a cylindrical shape like that of *S. Minnesota*. This hypothesis was further supported by the observation that variations in lipid A structures can impact the LPS capacity to activate TRPA1 (in vitro) and to cause inflammation (in vivo). Overall, it was shown that LPSs with asymmetrical hexa‐acylated lipid A (like *E. coli*, *S. typhimurium* and *Klebsiella pneumoniae*) induce the strongest TRPA1 responses, while LPSs with asymmetrical penta‐acylated lipid A (like *Serratia marcescens* and *Pseudomonas aeruginosa*) (Figure 1) trigger minor effects on TRPA1. Finally, LPS from *Neisseria meningitidis*, with a symmetrical hexa‐acylated lipid A, like that from *S. Minnesota* had no effect on TRPA1. Consequently, given the considerable heterogeneity of lipid A among various bacteria, further investigation of the precise molecular mechanisms underlying how the diverse LPS structural features influence interaction and activation of TRPA1 is compulsory.

Another TRP channel that can be activated by LPS is TRPV1 (Figure 6).^[77]^ TRPV1 primarily appears to have a pro‐inflammatory role in inflammation and sepsis. However, it has been also reported about an anti‐inflammatory effect on the spleen.^[83]^ Of note, the absence of TRPV1 in TRPV1 KO mice resulted in an increase of macrophages, neutrophils, DCs, and CD4 T cells in the spleens compared to wild‐type mice after administration of S‐LPS from *E. coli* O111 : B4 (Figure 3).^[84]^ This effect is believed to be mediated by LPS‐dependent TRPV1 activation, which leads to the activation of the sympathetic nervous system or noradrenergic neurons.^[18]^ A recent study has shown that TRPV1 plays a pivotal role in LPS‐induced calcium influx with the abovementioned production of pro‐inflammatory mediators in BMDMs.^[85]^ It has been shown that challenge with *E. coli* 055 : B5 S‐LPS (Figure 3) induces phosphorylation of IkB and MAPK in wild‐type BMDMs but not in TRPV1−/− ones.^[83]^ Additionally, evidence indicated that TRPV1 mediates *E. coli* O111 : B4 S‐LPS (Figure 3) induced inflammation in human induced pluripotent stem cell‐derived cardiomyocytes (hiPSC CMs).^[86]^

Strikingly, a paper by Alpizar *et al*.^[87]^ examined the role of TRPV4 channel in response to *E. coli* 0127 : B8 S‐LPS (Figure 3) in airway epithelial cells. They found that TRPV4 can mediate LPS‐induced calcium responses in mouse tracheobronchial epithelial cells without involving TLR4. This sensing of LPS by TRPV4 resulted in a direct antibacterial effect via nitric oxide production and increased ciliary beat frequency. However, in marked contrast, a recent study by Wang *et al*.^[88]^ provided evidence that TRPV4 is not the sensor for LPS‐induced calcium signaling in human bronchial epithelial cells and mouse ear skin single‐cell suspensions demonstrating that *in vitro* effects induced by S‐LPS from several *E. coli* strains (0127 : B8, 055 : B5, 0127 : B8 and K235) (Figure 3) as well as *S. Minnesota* S‐LPS are independent of TRPV4. Additionally, the authors also showed that *in vivo* all the above S‐LPSs did not evoke significant difference in inflammation and pain hyperalgesia between wild type and TRPV4 deficient mice. Although extremely interesting, these studies only analyzed S‐LPS from *E. coli* and *Salmonella*, therefore additional studies are required to validate that LPSs from other bacterial species (i. e. with a completely diverse chemistry) do not directly activate TRPV4 channel.^[88]^ By contrast, other studies that investigated the ability of TRPV4 to detect LPS in the urinary tract showed that stimulation of human urothelial cells with *E. coli* LPS resulted in a TRPV4‐mediated increase in intracellular calcium, as also seen in mice.^[89]^ Conversely, S‐LPS from *K. pneumoniae* and *P. aeruginosa* produced a lower increase in intracellular calcium levels compared to *E. coli* S‐LPS, thus paving the way to future studies aimed at evaluating whether variations in the LPS chemistry differently impacts on TRPV4 activation in the urinary tract.^[89]^

Finally, other TRP channels that have been found capable of being activated by LPS are TRPM3 and TRPM8.^[90]^ Despite its ability to sense the presence of LPS, TRPM3 has been found to be less sensitive to *E. coli* S‐LPS than TRPA1 and TRPV1. In contrast, human TRPM8 appears to be insensitive to this LPS at physiological temperatures but interestingly it can sense LPS at colder temperatures (25 °C) in a dose‐dependent manner.^[89]^ While the involvement of TRP channels in LPS‐induced inflammation, immune responses, and pain is becoming increasingly evident, significant gaps remain in our understanding of the interaction(s) occurring between LPS, its highly diverse chemistry, and these channels. Therefore, inspired by the elegant study of Jin *et al* (2024),^[79]^ future research should focus on the detailed chemistry of LPS, particularly its lipid A structure, to better understand how these molecular differences influence TRP channel activation and contribute to the pathophysiology of sepsis and inflammation. Further studies are also required to clarify the specific roles of each TRP channel in different tissues and under varying conditions of (chemically different) LPS exposure.

## 5 Human Lectins‐LPS Interactions

Lectins are carbohydrate binding proteins that play an active role in the immune response, facilitating communication and regulation of immune cells.^[91]^ Due to their ability to recognize carbohydrate structures, they have been identified as possible receptors for the sugar portion(s) of LPS.[^92^, ^93^] However, also in this case, the molecular mechanisms underlying LPS recognition by human lectins are still understudied.[^94^, ^95^]

The family of C‐type lectin receptors is among the most extensively investigated ones, as they mediate the activation of antigen‐presenting cells and therefore are essential for adaptive immunity, homeostatic regulation, cytokine and antibody production, and in general for the organism defense.^[96]^ Dendritic cell‐specific intercellular adhesion molecule‐3‐grabbing non‐integrin (DC‐SIGN) is a C‐type lectin that facilitates phagocytosis of various bacterial strains by DCs, thereby contributing to host defense against pathogens.[^97^, ^98^, ^99^] This lectin exhibits selective recognition of oligosaccharides bearing mannose and fucose, which can be present in LPS structures, but exerting a divergent behavior: i. e. an anti‐inflammatory response to fucose‐expressing ligands and a pro‐inflammatory response to the mannose‐expressing ones.[^100^, ^101^] For instance, it was shown that the intracellular signaling pathways of DC‐SIGN and TLR4 converge in response to fucose‐containing LPS from *Helicobacter pylori* (Figure 3), a spiral‐shaped bacterium that can chronically colonize the human gastric mucosa, resulting in a clear shift of T‐cell immune response.[^102^, ^103^] Briefly, it is typically observed a shift from Th1 to Th2, that is from a pro‐inflammatory to an anti‐inflammatory response, that facilitates the persistence of *H. pylori* infections. Mimicking the extraordinary complexity of *H. pylori* LPS chemistry, it might be speculated that also harmless commensal bacteria residing our intestines, such as those considered the normal human gut microbiota, decorate their LPS with sugar units that might be sensed by DC‐SIGN (and/or other lectins) leading to immune tolerogenic responses thus enabling their resilience within the host. In this frame, binding studies on *ad hoc* synthesized derivatives of LPS from *B. vulgatus* mpk have highlighted that fucose and the directly linked galactose residues, as well as galactofuranose and Kdo, are involved in LPS recognition by DC‐SIGN.^[104]^ In particular, fucose was shown to be crucial in this interaction, as it is located in the protein binding site where it acts as an anchor to DC‐SIGN. In addition, it was proposed a complex binding epitope for DC‐SIGN that involved both the core and the O‐antigen of *B. vulgatus* LPS, i. e. the terminal α‐fucose located in the inner core portion and a terminal β‐mannose in the O‐antigen.^[104]^ Overall, it was hypothesized that the concomitant presence of mannose and fucose in *B. vulgatus* LPS structure might contribute to the establishment of gut immune homeostasis thanks to this interaction with DC‐SIGN which in turn enables for a balanced host immune reaction.^[104]^ However, it should be underlined that, in the last decades, DC‐SIGN was shown to also recognize other sugar residues[^105^, ^106^, ^107^, ^108^, ^109^, ^110^] A combination of techniques, including NMR and molecular dynamics, has recently been employed to study the interaction between *E. coli* R1 core OS (Figure 3) and DC‐SIGN.^[111]^ The studies demonstrated that DC‐SIGN binds to the deacylated *E. coli* R1 R‐LPS (Figure 3) primarily through the recognition of its outer core pentasaccharide, which acts as a cross‐linker between two distinct tetrameric units of DC‐SIGN. Likewise, *N. meningitidis* can interact with DC‐SIGN in a manner dependent on the molecular composition of its LPS with important consequences on adaptive immunity. Indeed, by evaluating a panel of *N. meningitidis* LPS oligosaccharide mutants for their DC activating potential, Steeghs *et al*. (2006)^[112]^ discovered that *N. meningitidis lgtB* R‐LPS, expressing a GlcNAc(β1‐3)‐Gal(β1‐4)‐Glc outer core is targeted by DC‐SIGN thereby causing increased bacterial uptake by DCs and skew of the immune response into a T_H_1 direction.^[112]^ Overall these findings point out the urgent need for a comprehensive understanding of the interaction(s) between DC‐SIGN and the diverse LPS structures within our body; once again shedding light on this is crucial for the development of efficacious drugs and immunostimulant therapies against bacterial infections and immune‐mediated diseases.

Other widely investigated lectins, such as galectins, have been shown to recognize LPS.[^113^, ^114^, ^115^] Galectin 3 (Gal‐3) is the most extensively studied in this sense and is known to bind two different LPS sites: the galactose in the carbohydrate portion and the lipid A domain,^[116]^ with the consequent modulation of the signaling caused by the LPS itself. A recent paper by Pirone *et al*.^[117]^ showed that Gal‐3 recognizes LPS from *P. aeruginosa* and might amplifies caspase‐4/11 activation and oligomerization, leading to enhanced pyroptosis. However, as pointed out by the authors, the data collected pertains to the LPS from *P. aeruginosa* and does not rule out the possibility that similar experiments conducted on LPS from different sources could yield different and/or conflicting results. Therefore, further studies are necessary to gain a deeper understanding of the role of Gal‐3 (and other galectins) in the activation of the non‐canonical inflammasome pathway and, more in general, its impact on LPS‐mediated immune response. Likewise, Mannose‐binding lectin (MBL) has been studied for its capability to interact with LPS. For instance, it has been shown that the core OS of the LPS from *Hafnia alvei*, a human opportunistic pathogen, is recognized by both human and murine MBL.[^118^, ^119^] Of note, performing screening analysis with different core OS regions from numerous opportunistic pathogens, it has been shown that, beside GlcNAc, heptose residues in the inner core region are the key epitopes detected by MBL.^[118]^ In contrast, despite its *manno*‐configuration, Kdo residues are unlikely to serve as MBL ligands, as mutants expressing LPS consisting only of lipid A and one, two or three Kdo residues were not recognized by this lectin.[^118^, ^120^] However, the core OS accessibility may rely on natural LPS heterogeneity (i. e. coexistence of R‐LPS and S‐LPS in smooth strains), and is hindered by core OS substitution with the O‐antigen portion.^[118]^ Clarifying the specificity and affinity of MBL could enhance our understanding of the role of LPS chemistry in Gram‐negative infections overall, including those that result in sepsis or endotoxic shock. Finally, another human C‐type lectin known to interact with LPS is macrophage galactose‐type lectin (MGL), which typically plays an immunomodulatory role although studies on various cancer cell lines indicated MGL as a promoter of tumor malignancy.^[121]^ MGL was shown to bind to the terminal galactosides of the core OS of *Campylobacter jejuni* and *E. coli* R1 R‐LPS (Figure 3).[^122^, ^123^] It is worth noting that, only the affinity of MGL to LPS lacking the O‐antigen has been explored so far; however, most clinically relevant Gram‐negative bacteria possess this sugar domain. Therefore, future studies to better understand how and if MGL detects full‐length LPS and the implications in the regulation of the immune response are required.

Sialic acid‐binding immunoglobulin superfamily (Siglecs) represents one of the most studied lectins and nowadays they are acknowledged as glyco‐immune checkpoints in the field of cancer immunotherapy.[^124^, ^125^] Given their affinity for sialic acid, sialylation of LPSs makes them an important binding target of Siglecs. Macrophages and DCs express Siglec‐7, and it has been demonstrated that this protein interacts with *Fusobacterium nucleatum ssp. animalis* ATCC 51191 via the mediation of its LPS.[^126^, ^127^] At the molecular level, Saturation Transfer Difference NMR studies have revealed that binding occurs mainly through terminal sialic acids but it is likely that other O‐antigen sugars also contribute to this interaction. Siglec‐1 and Siglec‐5 have been shown to bind the sialylated LPS of *N. meningitidis*,^[128]^ and several Siglecs interact with *C. jejuni* strains expressing mono‐ and di‐sialylated R‐LPS.[^129^, ^130^, ^131^] Strikingly, binding of Siglec‐7 has been shown to correlate with its ability to induce anti‐GQ1b antibodies and is associated with Guillain‐Barré syndrome.[^132^, ^133^] Another example of R‐LPS with a terminal residue of sialic acid is *Nontypeable Haemophilus influenzae* (NTHi), which interacts with Siglec‐14 with the consequent increase of the production of inflammatory cytokines, which is related to exacerbation of Chronic Obstructive Pulmonary Disease.^[134]^

## 6 HMGB1 Enhances LPS‐Induced Inflammation

**Figure 7:**
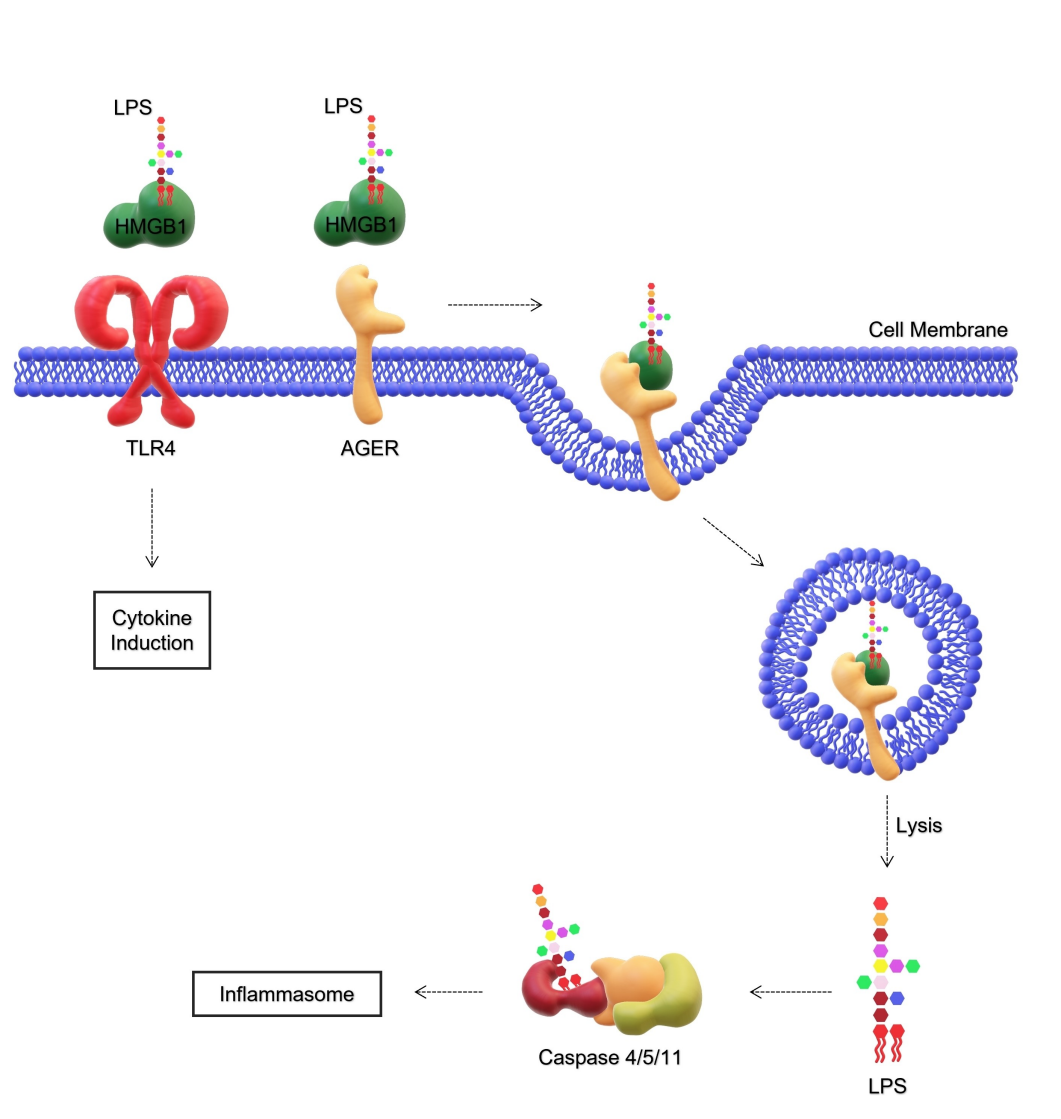
HMGB1 can activate different receptors in collaboration with a range of immune mediators. HMGB1 can form an extracellular HMGB1‐LPS complex by interacting with both the lipid and saccharide moieties. LPS can be presented to the MD2/TLR4 complex to follow the canonical TLR4‐dependent pathway or to AGER through which it is endocytosed. Through the endosome‐lysosome mechanism, LPS is released into the cytosol and is free to follow the caspase pathway, ultimately leading to inflammasome activation. Not drawn to scale. Abbreviations: LPS, Lipopolysaccharide; AGER, Glycosylation end product specific receptor; HMGB1, High‐mobility group box 1; TLR4, Toll‐like receptor 4

High‐mobility group box 1 (HMGB1) is a highly conserved nuclear protein in mammals,^[135]^ that plays essential roles not only in the nucleus of eukaryotic cells, but it also performs numerous functions in the extracellular space.^[136]^ Indeed, it is released when cells are activated or undergo necrosis, acting as a damage‐associated molecular pattern (DAMP), thus mostly with the intent to implement inflammatory functions. Its presence in the extracellular area, in fact, stimulates cytokine production and activation of both dendritic and T cells.[^137^, ^138^] In this frame, HMGB1 can greatly amplify the response elicited by LPS. This synergistic work is due to HMGB1 ability to bind several receptors, including TLR4 and the receptor for advanced glycation end products (AGEs), as well as TLR2, TLR9, and others (Figure 7).[^139^, ^140^, ^141^, ^142^, ^143^] In particular, it was hypothesized that LPS needs HMGB1 to trigger fulminant inflammation.^[142]^ HMGB1 activates the MD‐2/TLR4 through its association with MD‐2 that results in enhancement of signaling cascades and therefore production of pro‐inflammatory cytokines.^[144]^ Other components participating in this game are AGEs, for which HMGB1 has a strong affinity. In fact, addition of AGEs to the LPS‐HMGB1 mixture synergistically potentiates TNF‐α expression in macrophage‐like cells via mediation of TLR4 and the receptor of AGEs.^[145]^ Specifically, LPS is transported into the cytosol through the AGE receptor, facilitated by HMGB1, which then activates the caspase 11‐dependent inflammasome (Figure 7).^[146]^ HMGB1 has also been observed to facilitate LPS transfer to CD14 and, consequently, to TLR4 for downstream signaling, leading to NF‐κB activation, TNF‐α production, resulting in an enhanced inflammatory response.[^147^, ^148^]

Under a structural perspective, the interaction between HMGB1 and S‐LPS from *E. coli* O111 : B4 (Figure 3) has been described by Youn and coworkers and occurs through two LPS binding motifs for lipid A and the O‐antigen located in two different domains of the protein.^[147]^ The interaction of two synthetic peptides containing these motifs resulted in the inhibition of LPS‐stimulated TNF‐α production, indicating that both the lipid and saccharide portions of LPS are involved in the binding. Briefly, one of the HMGB1‐derived peptide (HPep6) showed to interact ionically with the phosphate‐head groups of lipid A.[^147^, ^148^, ^149^] In contrast, the other peptide (HPep1) binds to the polysaccharide moiety of LPS. Therefore, peptides targeting these binding sites could serve as potential therapeutic agents for sepsis, enhancing survival rates in conditions driven by LPS‐mediated sepsis.

Overall, there is clear evidence of an interaction between LPS and HMGB1 in the pathogenesis of Gram‐negative sepsis. However, despite attempts to neutralize LPS in clinical studies, no significant improvements in HMGB1‐driven sepsis outcomes have been achieved. Additionally, no research has specifically targeted HMGB1 or explored the potential impact of the highly variable LPS chemistry on these processes.

## 7 BAI1 Recognizes LPS

**Figure 8:**
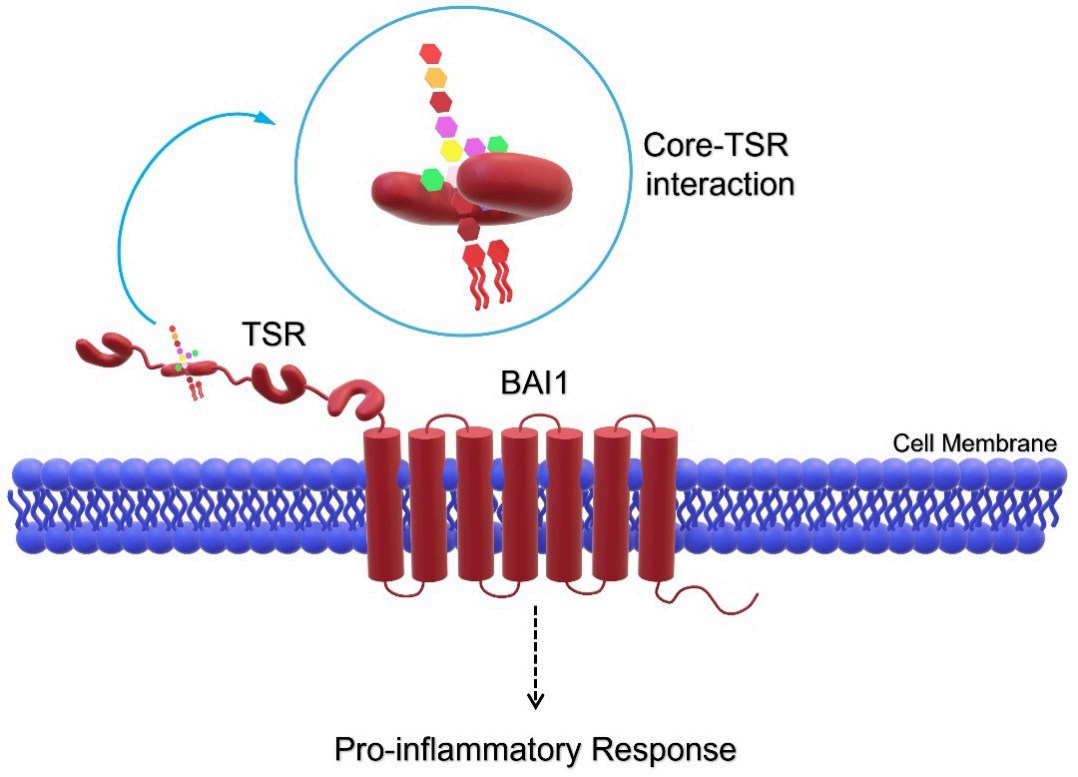
Brain angiogenesis inhibitor 1 (BAI1) uses its thrombospondin type 1 repeat (TSR) domains to interact with LPS mainly in the core OS region triggering a pro‐inflammatory response. Not drawn to scale.

Brain‐specific angiogenesis inhibitor 1 (BAI1) is the most studied member of the family of G‐protein‐coupled adhesion receptors (GPCRs), characterized by a multi‐domain structure and a size of approximately 200 kDa.^[150]^ BAI1 was initially linked only to the brain system due to its high presence in neurons and glial cells. However, it is currently known that it is expressed in a broader range of cell types and is engaged in a multitude of functions, including the clearance of apoptotic cells by macrophages.[^151^, ^152^] One of the most conserved and important regions of BAI proteins is the thrombospondin type 1 repeat (TSR), consisting of approximately 60 amino acids, which is present in several mammalian proteins and is known for its anti‐angiogenic properties.^[153]^ TSRs have been demonstrated to bind peptidoglycan, LPS, and lipoteichoic acids with varying degrees of specificity.[^154^, ^155^, ^156^] The ability of BAI1 to directly bind LPS via its TSR domains makes it crucial in the recognition of Gram‐negative bacteria (Figure 8). Indeed, the overall negative charge of *E. coli* 055 : B5 S‐LPS (Figure 3) was found to be essential for interaction with the conserved domains of TSR that expose numerous positively charged residues. Although distribution of charged residues varies greatly over the five TSR domains of human BAI1, both individual and in tandem TSR domains have shown the ability to bind *E. coli* LPS in protein array assays. Of note, structural mutation studies were also conducted with the objective of elucidating the residues most involved in the interaction process. For instance, the mutation of residues R284 and R307 in TSR1 resulted in the loss of ability to recognize this S‐LPS.^[157]^

In this frame, Das and co‐workers studies were of great significance, not only for the discovery of the direct interaction between LPS and BAI1, but also for the acquisition of preliminary information regarding the sections of LPSs that may exert the greatest influence in the binding event.^[150]^ Experiments in which an *E. coli* strain (DH5α) lacking the O‐antigen was used revealed that it was still able to bind BAI1, thus indicating that the O‐antigen is not a prerequisite for this interaction. By contrast, LPS variants containing or lacking the charged core OS demonstrated that the latter facilitates the interaction with the TSR domains of BAI1.[^150^, ^156^] This aspect once again underscores the significance of studying how and if this interaction occurs with chemically different LPSs. Accordingly, it has been recently shown that BAI1‐mediated recognition of LPS needs phosphorylation of the inner core heptose, thereby underlining the significance of electrostatic interactions in this process.^[158]^ Contextually, while the outer core appears to be unnecessary, the inner core part seems essential for interaction with TSRs. A more detailed study of the orientation of the residues could provide further insights into the relevance of phosphorylation (or other negative charges) within the core OS as well as the role played by sugar residues characterizing different LPS structures. Moreover, these findings indicate that BAI1 binds to a region of LPS other than lipid A, which is instead recognized by TLR4, possibly acting on a parallel or a convergent pathway. Accordingly, it was observed a decreased TNF‐α production after LPS treatment on macrophages deprived of BAI1, thus demonstrating that this protein may cooperate with TLR4 for an efficient pro‐inflammatory signaling.^[150]^ However, the mechanisms of this crosstalk remain to be determined.

## 8 Summary and Outlook

Being one of the main keywords in the language between host and microbes, LPS centered research activities are far to reach an end. Rather, innovative and unexpected avenues of exploration are looming on the horizon opening to intriguing opportunities of new discoveries that can potentially have repercussions in all fields of life sciences. The discovery of diverse, and sometimes concomitant, LPS recognition systems has tremendously advanced our understanding of the actual impact this molecule has in the dialogue with the host. Since 1998, when the TLR4 was identified as the immune sensor of LPS, we had clear evidence of numerous systems able to perceive and bind this glycoconjugate and, as such, taking place in this intricate game. These systems are both intracellular and extracellular, interacting or not each other, and, in a synergistic or antagonistic manner, are responsible for the (immune) response that our body produces when it encounters LPS. Complicating matters further, like in the case of TLR4/MD‐2, almost all (if not all) these newly discovered interactions seem to strongly depend on the chemical structure of LPS. This obviously raises important questions about the effects of the variations among the diverse LPS structures on these recognition systems and underlines how far we still are from a comprehensive understanding of these phenomena. On the other hand, it highlights that expanding our knowledge of the complex network of LPS sensing pathways paves the way for design, optimization and biomedical applications of these glycoconjugates, their synthetic derivatives and/or competitors for receptor binding, for translational research and benefit of human health.

All these recognition systems enable the host to promptly detect, respond, and clear the LPS‐expressing pathogens. Nevertheless, hyperactivation of these pathways, might result in multiorgan failure and death without any possible medical intervention, as is the case of sepsis. By contrast, they can be involved in the still poorly explored elicitation of tolerogenic mechanisms towards commensal and beneficial bacteria residing our body thanks to recognition of specific epitopes found in their LPS structures.[^1^, ^52^, ^159^, ^160^] Needless to say that gaining molecular insights into how this vital discrimination occurs is of enormous importance for the medical and biomedical field. As example, although still controversial, increasing evidence suggests that the recognition of LPS by lectins is critical in discriminating between harmful and beneficial interactions occurring within the host. Intensive research on the influence of LPS structural variations in binding and subsequent immune signaling is therefore urgently needed. Even more interesting would be to provide clear proofs of a crosstalk between lectins and other LPS sensors, such as MD‐2/TLR4 or caspases, and how this dialogue enhances/modulates immune responses. Indeed, although rationally intuitive that no single LPS sensor is the sole activating the immune response, information is entirely missing about collaborative mechanisms and dialogue between these multiple LPS receptors and the influence of LPS chemistry in these events. In addition, although some LPS recognition systems, like caspases and CD14/NFAT mediated pathways, have been investigated more than others, still many questions remain open and in most of the cases a tremendous lack of structure to function relationship data precludes us from using these glycoconjugates for immunity‐based therapies and/or next‐generation antibiotics. As example, the discovery that LPS requires the assistance of HMGB1 to induce severe inflammation has led to positive therapeutic results in preclinical studies of Gram‐negative sepsis targeting HMGB1.^[161]^ However, to date, no clinical trials have been conducted using this approach and lack of analysis aimed at unravelling the molecular details by which this interaction occurs limits the use of these mitigation measures. Likewise, BAI1 seems to play an active role in the regulation of TLR4‐mediated inflammatory signaling, but the mechanisms underlying this crosstalk and how the production of pro‐inflammatory molecules is initiated upon interaction with LPS still remains obscure. Further research is required to gain a comprehensive understanding of the role of TRPs in pathogen recognition and LPS detection. While most studies focus on the respiratory tract, it is crucial to investigate whether LPS interacts with TRP channels in other organs and tissues, and to appreciate what impact these interactions have on various pathological and/or physiological conditions.

Overall, we strongly deem that the exploration of the molecular aspects at the basis of these TLR4‐alternative/supportive LPS recognition systems should go together with the evaluation of how and if these sensing events are altered/modulated by the structural variability of LPSs, as immunological properties of these glycomolecules cannot be separated from their chemistry.[^1^, ^3^, ^162^, ^163^, ^164^] With this review, therefore, we intended to boost the interest of researchers of the medicinal chemistry and immunology field in the comprehension of the intricate network of LPS immune sensors by always using a structure to function approach. Only once a sound knowledge of how the structural features of LPSs affect the activity and dialogue between these detection systems, it will be possible to develop new drugs that mimic or antagonize their effects and to conceive novel strategies for treatment of immune‐related pathologies.

## Conflict of Interests

The authors declare no conflict of interest.

## Biographical Information

*Marcello Mercogliano is a post‐doctoral Research Grant Holder in Prof. Di Lorenzo group at the Dept. of Chemical Sciences University of Naples Federico II. He awarded the title of PhD in Chemical Sciences at University of Naples Federico II. He has demonstrated skills in organic chemistry synthesis as well as NMR and mass spectrometry‐based methods applied to carbohydrate and glycolipid chemistry. His research aims at elucidating the structural features at the basis of the immunomodulatory properties of bacterial glycans and glycoconjugates while exploring their complex structure‐function relationship*.

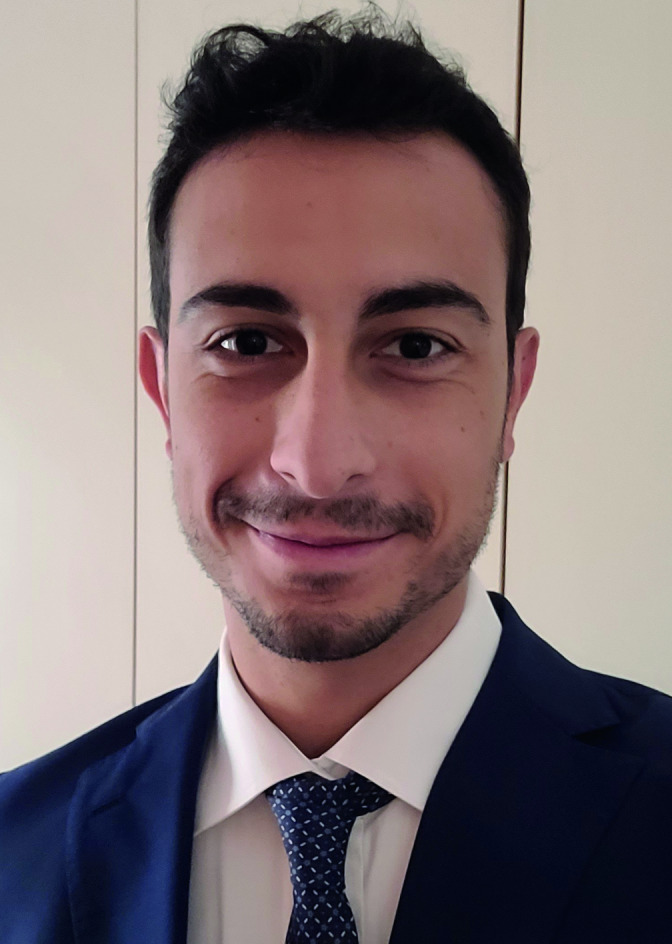

## Biographical Information

*Flaviana Di Lorenzo is Associate professor at the Dept. of Chemical Sciences University of Naples Federico II and CEINGE. She is an ERC Starting Grant awardee and PI of other National and International projects. She is the winner of the Nowotny Award 2024 bestowed upon a young investigator who has shown excellence in research, made a significant contribution to the study of endotoxins. She is an expert in chemical methods, NMR spectroscopy and HR mass spectrometry in structural glycobiology. She also has expertise in immunological techniques to assess glycans activity. She published more than 70 papers and leads a group of 7 people (4 PhD students and 3 post‐docs)*.